# A Systematic Review of Epoxidation Methods and Mechanical Properties of Sustainable Bio-Based Epoxy Resins

**DOI:** 10.3390/polym17141956

**Published:** 2025-07-17

**Authors:** Manuel Álvarez, Anthony Reilly, Obey Suleyman, Caleb Griffin

**Affiliations:** 1 Departamento de Tecnología de la Edificación, Escuela Técnica Superior de Edificación, Universidad Politécnica de Madrid, Avda Juan de Herrera, s/n, 28040 Madrid, Spain; 2 Advanced Materials Research Laboratory, University of Strathclyde, Glasgow G1 1XJ, UK; anthony.reilly.2017@uni.strath.ac.uk (A.R.); obey.suleyman.2017@uni.strath.ac.uk (O.S.); caleb.griffin@strath.ac.uk (C.G.)

**Keywords:** systematic review, epoxidised vegetable oils, mechanical properties, epoxidation process

## Abstract

There has been a growing interest in polymer-based materials in recent years, and current research is focused on reducing fossil-derived epoxy compounds. This review examines the potential of epoxidised vegetable oils (EVOs) as sustainable alternatives to these systems. Epoxidation processes have been systematically analysed and their influence on chemical, thermal, and mechanical properties has been assessed. Results indicate that basic, low-toxicity epoxidation methods resulted in resins with comparable performance to those obtained through more complex common/commercial procedures. In total, 5–7% oxirane oxygen content (OOC) was found to be optimal to achieve a balanced crosslink density, thus enhancing tensile strength. Furthermore, mechanical properties have been insufficiently studied, as less than half of the studies were conducted at least tensile or flexural strength. Reinforcement strategies were also explored, with nano-reinforcing carbon nanotubes (CBNTs) showing the best mechanical and thermal results. Natural fibres reported better mechanical performance when mixed with EVOs than conventional systems. On the other hand, one of the main constraints observed is the lack of consistency in reporting key chemical and mechanical parameters across studies. Environmental properties and end-of-life use are significant challenges to be addressed in future studies, as there remains a significant gap in understanding the end-of-life of these materials. Future research should focus on the exploration of eco-friendly epoxidation reagents and standardise protocols to compare and measure oil properties before and after being epoxidised.

## 1. Introduction

In the last decades, polymer-based materials experienced an increased presence due to their great versatility and performance in a wide range of industrial applications ranging from automotive to aerospace sectors [1]. At the same time, the production of polymer-based materials was mainly developed through fossil-derived products, thus significantly contributing to greenhouse gas emissions and environmental degradation [2]. Within these polymer-based materials, epoxy resin stands out due to its chemical stability, mechanical properties, low cost, and application possibilities [3].

Chemically, epoxy resins are three-membered cyclic ethers characterised by an oxygen atom bonded to two adjacent carbon atoms. This ring structure—referred to as epoxy groups, oxirane, or epoxide groups—typically formed by the oxidation of alkenes, is inherently strained, making epoxides highly reactive [4]. This reactivity is especially valuable in polymer chemistry, as it facilitates ring-opening reactions with various hardeners [5]. When incorporated into epoxy resins, these reactive groups enable the formation of a densely crosslinked, three-dimensional network, which is responsible for the resins’ excellent mechanical strength, thermal stability, and chemical resistance [6]. The unique molecular architecture of these resins not only facilitates a wide range of modifications but also improve their physical and chemical properties.

Despite their excellent performance and widespread use, most commercially available epoxy resins are still primarily derived from fossil fuels. Recently, increasing dependence on these non-renewable resources raised several concerns and challenges [7]. Fuel-based epoxy resins demand high energy consumption during production, thus significantly contributing to CO_2_ emissions. Even though epoxy resin systems led to a great technological development and broadened their applications, they also contributed to intensity pollution, resources, and energy consumption, thus presenting another challenge in end-of-life management [8]. Moreover, this elaboration also needs by-products that are not readily recyclable or degradable [9]. This has a significant impact on environmental sustainability and the lifecycle of these materials.

Consequently, researchers have been increasingly turning to sustainable alternatives to develop greener epoxy systems. Thus, in the last five years, there were several studies focusing on replacing traditional fuel-based compounds. In this sense, vanillin and lignin derivatives have been studied and proved as potential traditional fuel-based modifiers due to their renewable origins and lower environmental impact [10,11]. Specifically, vanillin’s aromatic structure results in greater rigidity and thermal stability to the resin, thus enhancing its mechanical properties and expanding its potential applications [12]. In a similar context, lignin derivatives are synthetised and used as the matrix for rein elaboration [13,14]. These alternatives demonstrate lower toxicity and greater biodegradability compared to conventional systems, thus setting new pathways to be explored [1]. Nevertheless, the use of these materials often requires further chemical modification to meet the high-performance requirements, thus reducing sustainability and functionality.

Among other bio-based alternatives, EVOs gained attention as a replacement for epoxy resin production. Vegetable oils (VOs), primarily composed of triglycerides, form when three fatty acid molecules combine with a glycerol backbone through esterification [15]. The fatty acids in these oils differ based on their degree of unsaturation—that is, the number of carbon–carbon double bonds present. This variation leads to the formation of saturated fatty acids (SFAs) such as palmitic acid, monounsaturated fatty acids (MUFAs) such as oleic acid, and polyunsaturated fatty acids (PUFAs) such as linolenic acid. Through a controlled epoxidation process (Prileschajew reaction) carbon–carbon double bonds can be converted into oxirane rings, thus making from them valuable intermediate products such as lubricants and plasticisers [16].

Consequently, numerous studies focused on the development of resins based on epoxidised oils, with the majority emphasising the epoxidation processes and the subsequent material characterisation [17,18]. Among these investigations, linseed and soybean oils are the most frequently employed, although less common sources, such as olive oil [19] and even recycled oils, have also been explored [20]. The specific conditions and methods of epoxidation significantly influence the final properties of the resins; even when the same type of oil is used [21,22]. Most studies primarily assess thermal and physical properties, such as glass transition temperature and thermal degradation temperatures, while other determinant data, such as mechanical strength and physical properties, are obviated [23,24]. Additionally, these works typically concentrate on evaluating the oils and resins themselves without advancing towards a defined application or end-use scenario [25].

The use of EVOs presents some challenges to be addressed; for instance, the dual nature of some vegetable oils. While many of them are non-edible and suitable for industrial applications, others compete with food resources. The potential application of edible oils for resin production could lead to an increase in market prices, thus setting up a potential economic dilemma [26]. Furthermore, the chemical structure of epoxidised vegetable oils significantly varies depending on the origin, affecting the chain length and degree of branching. This composition will significantly influence the efficiency and final properties of the cured resin.

Through a systematic review of the literature, this study aims to provide a comprehensive vision of epoxidised vegetable oil and its potential application in bio-based thermosetting resins. This paper systematically classifies the epoxidation processes, evaluating their complexity through a scoring system. Mechanical, chemical, physical, and thermal properties are gathered and analysed through the different studies conducted. Research trends are identified using bibliometric data to highlight areas of strength and gaps within the current literature. This analysis is expected to provide a solid foundation for the optimisation of bio-based epoxy resins.

## 2. Methodology

A comprehensive systematic review was undertaken within the domains of material science and sustainable polymer chemistry, with specific emphasis on the development, epoxidation processes, and mechanical performance of bio-based epoxy resins derived from vegetable oils. Following the PRISMA guidelines (Preferred Reporting Items for Systematic Reviews and Meta-Analyses), this study was carefully designed to ensure rigour, reproducibility, and transparency [27]. The aim was to critically assess recent advancements in the field, particularly in relation to mechanical performance, epoxidation complexity, and the chemical sustainability of reactive compounds used in resin synthesis.

### 2.1. Introduction to the Research and Research Question

As the polymer industry pivots toward more sustainable alternatives, epoxidised vegetable oils (EVOs) emerged as promising candidates for replacing conventional fossil-derived epoxy systems. While much has been reported on their chemical modification and epoxidation methods, less attention has been given to the integration of these green systems into high-performance resins. Thus, the overarching research questions guiding this review are as follows:

- What epoxidation methods are most commonly used to produce epoxidised oils for resin applications, and how do they compare in terms of complexity and sustainability?
- How do different curing agents, catalysts, and additives influence the mechanical and thermal performance of EVO-based resins?
- Which formulations offer the best balance between mechanical strength and environmental sustainability?

This study aims to clarify how the selection of oils, epoxidation processes, and curing systems affects both performance and ecological viability, while highlighting gaps and future opportunities for innovation.

### 2.2. Search Strategy, Inclusion and Exclusion Criteria

A structured search strategy was implemented using three major scientific databases: Scopus, Web of Science, and Springer. The following keywords and Boolean combinations were used: “epoxidation,” “bio-based resin,” “epoxy resin,” “vegetable oil,” “mechanical properties,” “epoxidized oils,” “thermoset,” “curing agent,” “thermal degradation,” and “green chemistry.” Additional search terms, such as “reinforcement,” “hardener,” “Tg,” “OOC,” and “crosslinking”, were included to refine results and capture studies that reported critical mechanical and thermal data.

The review was limited to peer-reviewed journal articles published between January 2020 and March 2025, capturing the most recent research efforts. Studies were included based on the following criteria:

- Focused on epoxidation or synthesis of thermosetting resins from vegetable oils;
- Included thermal data such as glass transition temperature (Tg) or degradation temperature (T_5%_);
- Used curing agents or catalysts whose identity was clearly described;
- Written in English.

Exclusion criteria included the following:

- Reviews, patents, or non-peer-reviewed material.

Three sequential searches were performed in each database to reduce publication bias. Where duplicate entries were encountered, only the most comprehensive or up-to-date version was retained. Titles and abstracts were screened manually, followed by full-text analysis for inclusion eligibility.

### 2.3. Data Extraction and Categorisation

A standardised Excel database was developed to systematically extract relevant information from the final set of included studies. Extracted data included the following:

- Bibliographic information: title, authors, publication year, DOI, and journal.
- Resin composition: type of oil used, epoxidation method, oxirane oxygen content (OOC), epoxy equivalent weight (EEW), and iodine value.
- Synthesis details: stoichiometry, catalyst or curing agents used, and curing conditions (temperature/time).
- Mechanical properties: tensile strength, modulus, elongation at break, and flexural strength/modulus.
- Thermal properties: Tg, thermal degradation (T_5%_), thermal stability index.
- Sustainability considerations: source of reagents, toxicity classification, and energy intensity of the synthesis.

Furthermore, additives such as reinforcements (e.g., natural fibers, carbon nanotubes, and nanoclays) were also recorded and categorised by type and morphology (powder, fiber, and nano). For each resin system, its reinforcement, processing method, and mechanical output were correlated to determine trends in performance.

### 2.4. Epoxidation Complexity and Chemical Scoring System

To rank the sustainability and practical feasibility of the epoxidation processes, a five-level complexity classification system was developed. Each process was evaluated based on the following:

- Reagent toxicity and environmental hazard (e.g., sulfuric acid = high, citric acid = low);
- Process duration (less than 3 h = simple; more than 6 h = complex);
- Operational temperature (above 80 °C = energy-intensive);
- Reagent type and number (single-step vs. multi-step or DES synthesis).

Processes employing the traditional Prileschajew reaction using glacial acetic acid and hydrogen peroxide were typically ranked as level 1 or 2, while more elaborate syntheses involving toluene, epichlorohydrin, or deep eutectic solvents (DES) were ranked 4–5 due to higher resource consumption and potential environmental risks.

### 2.5. Mechanical and Thermal Performance Benchmarking

To ensure meaningful comparison, only samples reporting tensile strengths above 10 MPa were included in the primary analysis. While a few outliers with lower mechanical values were noted for completeness, the focus remained on potentially viable industrial applications. Mechanical performance was examined in relation to the following:

- Oil source (already epoxidised vs. in situ);
- Curing system (aromatic vs. aliphatic hardeners);
- Catalyst presence and identity (imidazole, 1-MI, BDMA, etc.);
- Reinforcement strategy (fiber, nano, and powder).

Thermal behaviour was examined primarily through Tg values and degradation temperatures (T_5%_). Correlations between OOC and Tg were also explored, as were cases of brittleness or rubbery texture based on crosslink density and formulation.

### 2.6. PRISMA Flowchart and Result Consolidation

Following PRISMA methodology, a flow diagram was developed to illustrate the article selection process, from initial identification to final inclusion. An overview of the selected articles was summarised in tabular form, including key resin characteristics and performance outcomes. These results form the foundation for the subsequent discussion section, where trends are analysed, and standout formulations are critically evaluated.

### 2.7. Study Risk of Bias Assessment

Although this review systematically screened and extracted data from a wide array of recent publications, a formal risk of bias assessment tool (e.g., ROBIS or Cochrane RoB2) was not applied. This decision was made due to the heterogeneity in study types and reporting quality across the selected literature. Nevertheless, a qualitative evaluation was conducted based on reporting completeness, replicability of methodology, and consistency of results. Future systematic reviews on this topic could benefit from implementing a structured bias assessment tool to increase methodological transparency.

## 3. Results

Table 1 presents data collected from the most relevant articles, which were selected based on their comprehensive reporting of both mechanical and thermal properties. Only studies presenting both mechanical properties and key thermal metrics, such as degradation temperature and glass transition temperature, were considered. Furthermore, references with a lower tensile strength than 10 MPa were discarded as values below this level are insufficient to offer practical applications.

### 3.1. Analysis of Research Lines

**Figure 1:**
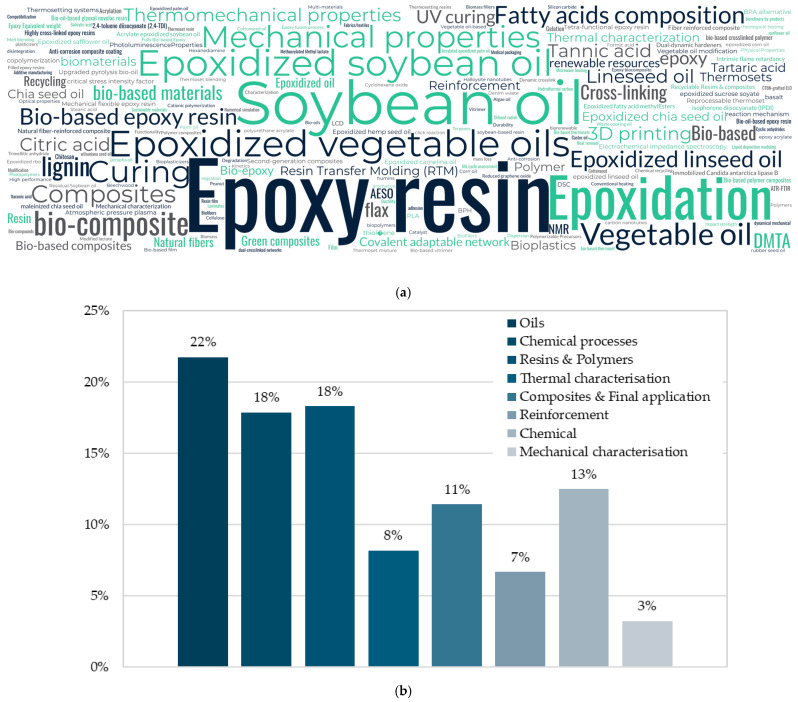
Keywords word cloud. (**a**) Keywords wordcloud; (**b**) percentage of keywords referred to each group.

Figure 1a shows keywords organised in a word cloud to understand their incidence from all the references consulted. On the other hand, Figure 1b shows how these keywords are distributed according to the categories established in the methodology.

This word cloud highlights the core themes and concepts explored across the literature. Across the references, “Epoxy resin” is the most used keyword. However, this generic term has been always linked to commercial/conventional fuel-based epoxy resins. According to these metrics, a new paradigm is arising as the bio-based resins are becoming widely studied over fuel-based. Then, authors tended to focus on the types of oils used and their natural origin. Soybean oil is the most used oil across the references, and thus, a quite common keyword. After that, “Vegetable oil,” “Linseed oil,” and “Epoxidized vegetable oils” suggest a broader discussion on bio-based feedstocks beyond just soybean oil. Terms such as “Bio-based materials,” “Green,” “Bioplastics,” and “Recycling” emphasise an eco-friendly approach, which means that research is focusing on sustainable alternatives to traditional fuel-based resins.

Regarding material characterisation, although keywords such as “mechanical properties”, “thermal properties”, and “curing” were widely used, most of the studies had a great lack of information on many of the usual properties that a study should comprise. It is true that keywords must express the general aspects of the paper, but most of the studies only focused on the elements used rather than the actual content of the paper. This could potentially mislead authors and researchers when trying to target information.

Figure 1b shows a distribution bar chart grouped in the topics explained above. It can be seen that most of the keywords used were related to oils (21.8% of the keywords). After that, chemical processes and resins and polymers were strongly used. Most common chemical processes were related to curing kinetics and crosslinking actions occurring during the study. After that, words indicate either the epoxidation or some modification processes needed to obtain the raw material. Keywords related to resins and polymer were mainly “Bio-based epoxy resin” “Epoxy resin” and “thermoset”. These studies focused not only on the epoxidation process, but also on the resin elaboration. It is worth it to note that mechanical characterisation-related keywords (4.3%) were the lowest found across the studies. This means that most of the studies did not provide meaningful results regarding the mechanical performance of the different resins. This is a promising gap considering that the studies have been found in several peer-reviewed journals.

**Figure 2:**
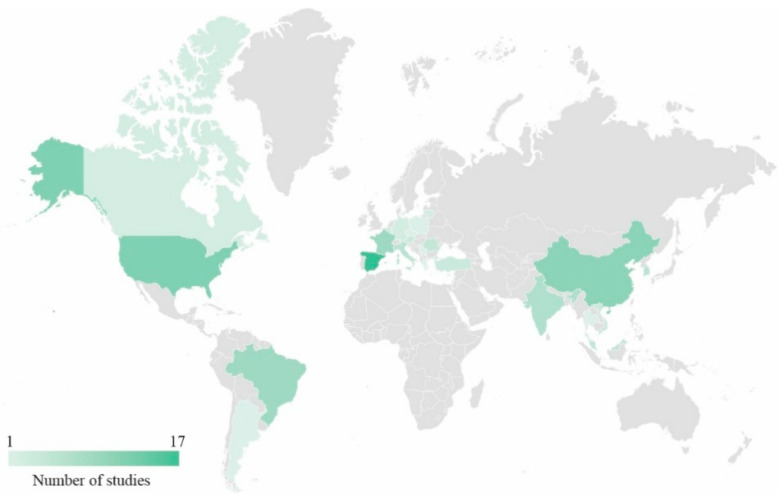
Origin of the studies analysed.

Last, from Figure 2, it is possible to picture origin countries where epoxidised vegetable oil studies were published. As can be seen, countries with modern scientific infrastructures (Spain, China, and US) and strong agricultural outputs (Brazil, India) lead the development and publication of bio-based material research. The low representation in other regions in South America and Europe highlights a potential gap and opportunity for future collaboration, thus expanding global expertise and innovation.

### 3.2. Vegetable Oils (VO)

To date, oils were already widely used in industry as precursors to develop new products such as cosmetics, lubricants [48], plasticisers, emulsifiers [49], and more recently, for thermoset resins [50]. Oil selection in EVO-based resins is determinant for the resin’s reactivity, curing behaviour, and thermal mechanical properties [51]. Within the studies consulted, 33 unique oils have been found, and either epoxidation, resin elaboration, or both were conducted on 120 oils, as several studies performed resin/epoxidation on distinct oils. Across the literature, a wide range of oils with distinct degrees of unsaturation and epoxidation have been found. These oils can be grouped by their origin as being seed-based, fruit-based, cereal/grain-derived, algae, wood-derived, shell, and others.

Seed-based oils are the most used across the studies (63.4%), ranging from commonly known soybean and linseed to the less used safflower [52,53], chia seed [54], and jatropha [55]. Their straightforward way to be obtained, together with their high availability are the main reasons to be studied. However, at this moment, most of them have a low productivity, thus making it more difficult to implement on a larger scale. Subsequently, fruit-based oil, such as palm oil, is also commonly studied [56,57]. Triolein oil obtained from algae [58], as well as wood-derived oils such as honokiol [59], has still an enormous potential to be deeply explored due to their availability and thermal stability. Cereal/grain-derived oils, such as rice bran, so far used with thermal insulation purposes [60], remain a great option to be explored in terms of epoxidation and resin production.

**Figure 3:**
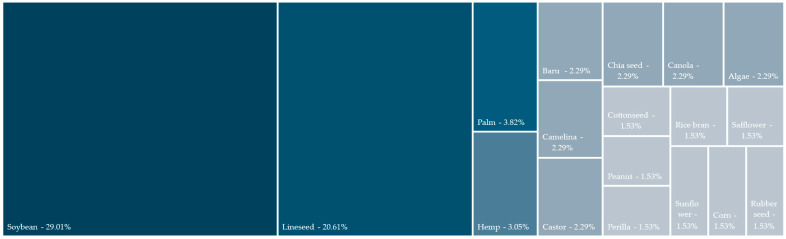
Oil use distribution.

Figure 3 shows the oil distribution across the studies analysed, appearing in at least two different studies. In total, 33 unique oils were used, and studies were conducted on 120 oils, as several studies performed resin/epoxidation on distinct oils. Half of the studies analysed used either soybean (30.8%) or linseed oil (20.8%) as a raw material for epoxidation/resin elaboration. Oils from palm and hemp (4.2% each) were the most common after them. Regarding the potential use of the oils across the literature, 55% of the oils used were edible oils, although common edible oils, such as olive [19] and sunflower oil [61,62], were not widely studied as their use could eventually lead to an increase in market prices. In total, 16 distinct types of oil were used once, highlighting Karanja [29], tung [61], and cardanol [63].

Across the literature, 44% of the studies selected already epoxidised soybean/linseed oils as raw material. This means that some of their compositional properties are standardised, or at least not influenced by potential differences during epoxidation processes.. From those whose epoxidation was conducted in situ, characteristics are quite different depending on the process complexity reagents used and epoxidation steps [39,64,65].

To classify their suitability for epoxidation and resin elaboration, parameters such as degree of unsaturation (iodine value IV) and either epoxidation OOC or EEW are mandatory to know, whereas fatty acid profile, viscosity, and molecular weight and hydroxyl value are relevant and could help to obtain a comprehensive understanding of both process and results. In this review, IV, EEW/OOC values have been gathered and analysed, and they are shown in Table 1. Only three studies showed compositional values for all the parameters mentioned [30,54,66]. IV—a key parameter to determine the amount of available double bonds regarding the epoxidation [67]—is mentioned in only 20% of the studies. EEW (33%) is determinant of establishing the resin-hardener stoichiometric ratio. This will ensure a correct curing process, influencing resin’s mechanical performance. Last, OOC is the most presented value (61%). Tough, already epoxidised oils are more likely to include this information, and values are not given equally in all studies. Despite the fact that OOC values can be similar, most of them vary from one study to another even in studies using the same oil [17,68]. Despite the information found in the literature, there is a lack of consistency in reporting these values. This triggers a great challenge when trying to establish comparisons and conclusions about the best choice for conducting epoxidation processes and resin development.

**Figure 4:**
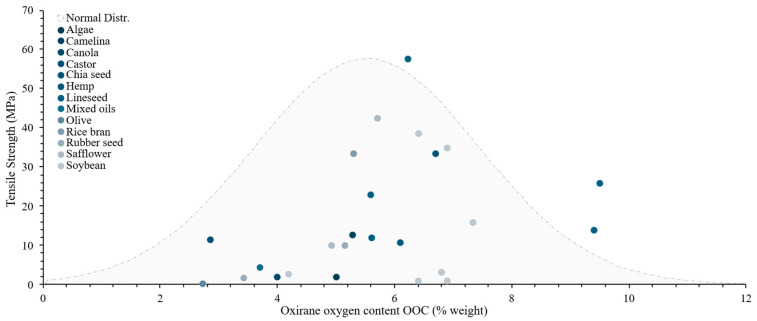
Tensile strength over oxirane oxygen content dispersion chart.

Regarding these properties’ influence on resins developed, Figure 4 shows a dispersion chart on oxirane oxygen content over tensile strength. A normal distribution according to the gathered data was depicted to contextualise the influence of this property. As discussed, oxirane oxygen content reflects the number of epoxide groups available for crosslinking, thus directly influencing the mechanical properties [69]. Typically, a higher oxirane oxygen content enables enhanced crosslink density. Nevertheless, this performance is also influenced by both the epoxidation process and hardener/catalyst used. Thus, high oxirane content reported brittleness, as the network might become too rigid, reducing the resin’s toughness.

### 3.3. Epoxidation Process

**Figure 5:**
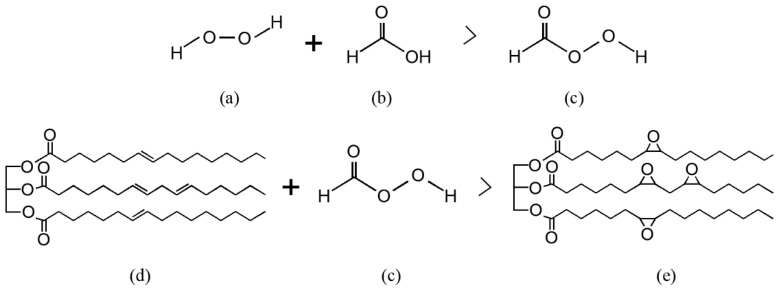
Prileschajew reaction process. Chemical structure of (**a**) hydrogen peroxide; (**b**) formic acid; (**c**) performic acid; (**d**) triglyceride molecule; and (**e**) epoxidised vegetable oil. Own elaboration.

The epoxidation process is mainly achieved through the Prileschajew reaction. This reaction involves converting alkenes into epoxides by peracid, typically performic acid (formic acid + hydrogen peroxide) or another peroxy acid as the oxidising agent. When mixed with the oil, peracid donates an oxygen atom to the alkene, thus forming a three-membered epoxide ring as depicted in Figure 5. This reaction is widely studied and recognised as the standard method to produce epoxidised vegetable oils [16].

The process works as follows: First, the acid is mixed with the oil and stirred to ensure a full solubilisation and homogeneous mixture. Then, hydrogen peroxide is slowly added dropwise and mixed again. Peracid adds an oxygen atom through epoxidation reaction to the double bond in the oil. Subsequently, the initial organic acid is regenerated, allowing it to be converted in peracid again in a cyclic process. Peracid formation is the rate-limiting step as it is a reversible reaction. Catalysts help to speed up this production. For this, H_2_SO_4_, HNO_3_, and H_3_PO_4_ are widely used across the literature [39,66,69,70]. This process varies depending on factors such as temperature or catalysts nature. Typically, this reaction’s temperature ranges from room temperature to 75 °C, with 60 °C being the most common temperature found across the studies analysed [70,71,72].

Epoxidation agents employed among all studies are shown in Table 2, where they have been rated 1–5 according to their disposability and environmental impact as follows: 5—Highly toxic, hazardous, and difficult to dispose of. 4—Corrosive or reactive but manageable with precautions. 3—Less toxic but still requiring careful handling. 2—Low toxicity and minimal environmental concerns. 1—Generally safe and environmentally friendly. Furthermore, chemical formulation, role within the epoxidation process, and reactivity are also shown to contextualise the current trends and main concerns about them.

From this table, it can be seen that there is a strong reliance on moderate-to-high-toxicity chemicals used across the literature, with hydrogen peroxide, glacial acetic, formic, and sulfuric acid being the most common reagents. These elements are highly reactive, thus requiring careful handling and treatment. Furthermore, they exhibit significant corrosiveness and environmental risk. Although more sustainable alternatives, such as Amberlite IR 120H—a relatively low-toxicity ion-exchange resin—is being increasingly used, it is not within the dominant choices.

On the other hand, agents such as epichlorohydrin, toluene, and phenol formalin solutions remain as the least implemented due to their serious health and environmental concerns. These solutions are carcinogenic and highly toxic. Though they are widely used, they are extremely corrosive and dangerous to humans and aquatic ecosystems. Even though low-toxicity solutions such as sodium salts and choline chloride are safer, they do not provide reactivities nor efficiency, thus making it more difficult to replace the use of these hazardous elements.

To offer a more systematic view, reagents were classified into five toxicity levels (from benign to highly hazardous), based on chemical structure, environmental persistence, and handling risk, following European CLP regulation principles.

Low-toxicity reagents such as acetylcholine, sodium bicarbonate, citric acid, and choline chloride (toxicity levels 1–2) stand out for their relatively benign environmental profiles. These compounds are biodegradable, originate from renewable or low-impact feedstocks, and are generally considered safe under most laboratory and industrial conditions. For instance, sodium bicarbonate, frequently used as a neutralising agent, offers a low-risk route for base-catalysed epoxidation, while choline chloride is often combined with natural acids to create deep eutectic solvents (DES), enabling greener solvent systems with minimised volatility and toxicity.

In contrast, reagents such as sulfuric acid, hydrogen bromide, and epichlorohydrin fall into the highest toxicity tier (level 5). These chemicals are highly reactive, corrosive, and environmentally persistent. Epichlorohydrin, a primary epoxidation agent in several studies, is derived from petroleum feedstocks and is classified as a probable human carcinogen (GHS H341, and H350), posing serious risks in both laboratory handling and downstream waste management. Similarly, sulfuric acid, one of the most widely used catalysts (13 occurrences), carries significant occupational hazards (H314), and requires extensive neutralisation and waste treatment procedures. Glacial acetic acid and hydrogen peroxide, although slightly less toxic (level 4), remain aggressive oxidants with the potential to generate hazardous reaction intermediates if improperly managed.

The overrepresentation of high-toxicity reagents in otherwise “green” resin formulations points to a critical contradiction in the field: while bio-based oils offer renewable carbon sources, their transformation often relies on non-renewable, energy-intensive, or hazardous chemicals. This diminishes the overall environmental benefit of the end product. As such, greater emphasis must be placed on optimising epoxidation protocols using low-toxicity catalysts and solvents. For example, replacing sulfuric acid with solid acid catalysts such as Amberlite IR 120H or employing biobased acids such as citric or oxalic acid (toxicity levels 2–3) could reduce environmental and health risks without compromising efficiency. Moreover, DES systems based on choline chloride and organic acids represent a promising frontier for solventless or recyclable green chemistry routes, albeit still underexplored at an industrial scale.

Ultimately, the sustainability of bio-based epoxy resins must be evaluated holistically—not only through their source materials, but also by considering the full lifecycle impact of each reagent used in synthesis. Advancing green chemistry in this field requires the concurrent reduction in hazard, waste, and energy input in epoxidation pathways.

**Figure 6:**
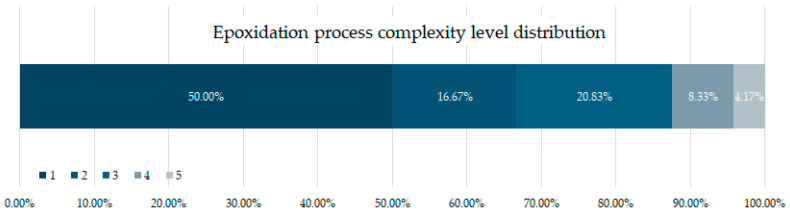
Epoxidation complexity level distribution.

Figure 6 shows the classification of epoxidation complexity levels that this review considered. First, 44% of the studies analysed in this review used already epoxidised oils. From the studies conducting epoxidation, 50% performed a basic Prileschajew reaction without further modification or minor modification, which gives them level 1 [73]. Epoxidation time remained below 3 h and temperatures below 60 °C. Across these studies, glacial acetic acid and formic acid are the most common acids used [18,56,71,74]. H_2_SO_4_ appears as a catalyst in most of the studies within this level, although commercial Amberlite IR120 (a less toxic catalyst) often replaces H_2_SO_4_ [75,76].

Epoxidation processes classified under complexity level 2 and 3 (16.67–20.83% of the studies) comprise reactions with higher temperatures (80–100 °C) and longer process times (6–8 h) required to achieve efficient conversion. Moreover, these conditions need to be monitored and controlled due to detonation risks. A longer reaction time and higher temperature translates into a higher energy consumption, thus, making the process less economically profitable and environmentally sustainable compared to level 1. Furthermore, auxiliary chemical agents such as toluene [77], photo initiators [36], and more toxic acids such as hydrochloric acid [78] not only increase the operational challenges, but also raise waste management, disposal, and potential environmental impact concerns. Finally, while these high-complexity methods may improve epoxidation efficiency, they also introduce significant technical and environmental trade-offs that must be carefully evaluated.

Epoxidation processes classified as complexity level 4 (8.33%) involve multiple reaction steps, such as several deionised water washes, and low-pressure evaporation to remove excess [59,79], elevated temperatures (>100 °C) [19], and the use of highly reactive chemical agents, making them significantly more challenging to implement on an industrial scale. These methods frequently employ epichlorohydrin [80], a highly toxic and reactive compound, alongside three or more auxiliary chemical agents. Additionally, longer processing times, intricate purification steps, and complex separation techniques contribute to increase operational costs and environmental impact.

At the highest level of complexity, epoxidation processes rated level 5 (4.17%) introduce an additional preparatory stage for epoxidation agents, further increasing the reaction time and resource consumption. These processes often involve the synthesis or modification of deep eutectic solvents (DES) [65], specialised catalytic systems [81], or customised peracids, which require pre-reaction steps before the actual epoxidation takes place. Moreover, the pre-requisite for multiple temperature variations across different reaction stages make the process less energy-efficient and harder to scale. Due to these steps, such methods are generally not considered suitable for large-scale industrial applications.

It also must be noted that most studies comprising complex epoxidation processes did not perform any type of mechanical testing [55,59,65,79]. However, other studies using vegetable oil reported similar values when both simpler and complex epoxidation methods were involved [47,82,83]. This suggests that even the most complex epoxidation methods do not mean a superior performance in the final EVO [39]. After considering this, the added effort, cost, and resource consumption associated with these processes may not be justified.

### 3.4. Resin Production Process

To develop resins from epoxidised oils, different reagents were used across the consulted literature. The basic elaboration process consists of mixing the reagents at different temperatures and pouring them into different shaped moulds. Afterwards, curing processes consisted of either placing them in an oven (setting different temperature and time) or submitting them to UV light curing [84]. As expected, both curing conditions and reagents used affect mechanical performance. To understand and picture the current trends on resin elaboration, reagents were grouped according to their origin in natural compounds, synthetic organic compounds, and inorganic catalysts and additives.

First, natural compounds such as maleinised oils, linseed [85], chia [54], and hemp [86] are used, although their application reported a significant negative impact on mechanical strength. Tannic acid is widely used [45,71,87] together with ethanol as catalyst. Though it has a high price and relatively moderate impact on the final properties, it is a reasonable option compared to other—more toxic—synthetic alternatives. Across the literature, natural compounds are not widely used despite the range of potential reagents available [1,14,88].

Subsequently, most used synthetic organic compounds are amines, acrylic compounds, and commercial epoxy hardeners. The use of commercial reagents, such as TMPTA [89], provides high crosslink density and excellent thermal stability. However, they raise toxicity concerns due to the presence of bisphenol A and TMPTA, which may induce brittleness and cost challenges. On the other hand, amine-based hardeners, such as cycloaliphatic amines (e.g., JONCRYL), DDM [90], and N,N-dimethylbenzylamine [45], accelerate curing and improve heat resistance. The main concerns are the potential health risks associated with their high toxicity. Finally, acrylic compounds—acrylic acid [40] and IBOMA [36]—influence parameters such as flexibility and impact resistance. Dicarboxylic/polycarboxylic acids such as itaconic, adipic [40], and citric [62] are less harmful to the environment, although their reactivity varies depending on the catalysts and oil used.

Despite the superior performance of synthetic catalysts—primarily derived from fossil fuels—in achieving robust curing kinetics and enhanced thermal stability, their environmental and health drawbacks are increasingly evident. In contrast, natural catalysts, such as deep eutectic solvents (DES—mixing organic acid-base pairs) and maleinised oils, although less explored, offer a less harmful alternative with significant potential. Consequently, advancing large-scale replication techniques and application-driven studies for natural compounds could pave the way toward more sustainable and commercially viable epoxy resin systems.

Finally, curing conditions—time and temperature—were studied and compared. The complexity of curing conditions is evident as the literature contains studies focused on this process, varying time and temperature to achieve the best performance [91,92]. While every study uses different conditions, tendencies were observed. Two different curing conditions are applied across the reviewed literature. Studies performing UV light curing were dedicated to 3D printing purposes [33,47,93]. Nevertheless, typical curing processes occur in an oven, with temperatures ranging from 80 to 170 °C and distinct conditions. Although most of the studies comprise a two-step curing process, there were also studies that increased it up to 4–7 times [19,20,24]. Despite this wide range of temperatures, mechanical properties are affected by reagents used and type of oil than by these conditions. Studies focused on curing conditions confirmed this, as higher mechanical strength values used similar curing conditions than studies that obtained lower mechanical strength values [19,61,94].

### 3.5. Characterisation Techniques

To understand how previous studies analyse the properties of both epoxidised oils and the resins derived from them, this review systematically compiled the tests performed in each study and classified them into the groups described in Section 2. A total of 64 unique tests were identified across the 85 articles reviewed. These tests have been grouped depending on their nature. Table 3 shows top ten tests performed and the tests’ topic distribution across the references studied.

Among these categories, the largest share of tests focuses on chemical (30.66%) and thermal (30.30%) properties, where FT-IR stands out, being reported in 74.42% of the papers, followed by NMR (40.40%). TGA, DSC, and DMTA appear in 68.60%, 62.79%, and 50% of the studies, respectively. Thermal conductivity and transmittance are also studied, as papers use these resins to coat cables. This parameter would also be interesting regarding resin’s applications to other fields, such as building. Morphological properties through SEM images (32.56%) are also included in the studies, though its presence is significantly lower than other properties.

Physical properties tests account for 18.72% of the total, although it is notable that something as fundamental as density appears in only 5% of the studies. By contrast, shore D hardness (16.38%), water contact angle, and water absorption (15.12% each) are the most frequently reported tests in this category. This category contains a wide variety of tests, although most of them are included in few studies. Surprisingly, essential information such as EEW or OOC and crosslink density remains underexplored. Most of the values for these tests are taken from already epoxidised vegetable oils. EEW is a key parameter to develop epoxy resins, as it is required for distinct stages of the elaboration, such as stoichiometric ratio, which will influence curing behaviour, and thus final mechanical performance. There is a severe lack of information within this category that must be considered. It is worth highlighting the low proportion of mechanical characterisation tests (10.78%) present across the literature. Moreover, just 38% of the publications conducted tensile tests, and only 19.77% included flexural tests. It is striking that studies perform both the epoxidation process and resin development, but do not assess the mechanical properties, despite these being crucial to set their potential applications.

**Figure 7:**
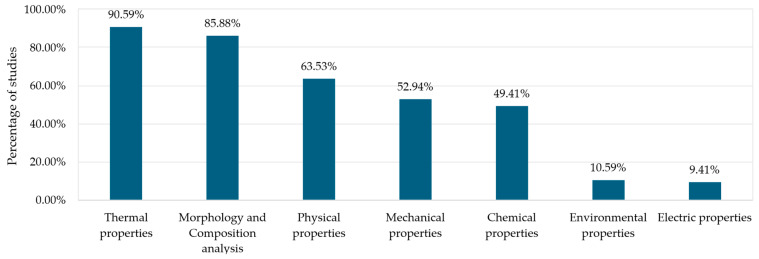
Tests topic appearances across the references.

Subsequently, Figure 7 highlights the frequency of tests performed among all considered studies. As expected, thermal and morphological/compositional analysis tests are performed in over 80% of the studies consulted. These tests are necessary to determine key parameters such as thermal stability and chemical structure. Moreover, the equipment required to assess thermal properties is quite common and available. Nevertheless, this high percentage does not mean that studies comprise a complete thermal characterisation. Most of the studies performed either TG, DSC, or DMTA, but only a few conducted the full thermal characterisation.

The marked difference between the abundance of thermal and morphological characterisation tests (primarily TGA, DSC, and FT-IR) and the relative scarcity of chemical and mechanical characterisation is especially striking. While thermal characterisation (e.g., TGA, DSC) provides valuable information on the material’s stability and behaviour under temperature changes, its relevance may be less critical than a more in-depth chemical characterisation—which, for example, confirms the efficiency of the epoxidation or the degree of crosslinking—and a comprehensive mechanical characterisation, which is essential for industrial applications.

It is surprising that, despite most studies focusing on oil epoxidation and resin synthesis, only around 50% include any type of mechanical testing, and in many cases, key parameters, such as the modulus of elasticity or elongation at break, are omitted. This lack of data hinders a realistic evaluation of the structural properties and strength of the resins, posing a challenge for validating their use as alternatives to commercial epoxy resins. Furthermore, the limited presence of detailed chemical tests (for instance, determining the epoxy equivalent weight or crosslink density) constrains the ability to correlate composition with mechanical and thermal performance.

This trend may be explained by the predominantly academic approach in many studies, which prioritises the exploration and optimisation of laboratory-scale synthesis and the characterisation of more basic properties. However, for these resins to be competitive in the market and replace conventional epoxy resins, it is crucial to conduct deeper investigations of their mechanical and chemical properties. Without these values, it is difficult to ensure the reliability and durability of the resins when applied. Therefore, future work should comprise comprehensive characterisation, including balanced thermal, chemical, and mechanical tests.

Finally, although a few studies include environmental assessments, only one provides a superficial life cycle assessment (LCA). This indicates that research efforts in this field remain strongly focused on enhancing material’s performance, leaving a significant opportunity for a more in-depth exploration of mechanical and environmental aspects. After this analysis, the pathways still to be explored are mechanical and environmental performance, thus helping researchers to check the feasibility of this alternative to conventional fuel-based epoxy resin, especially considering the European trend policies regarding the reduction in non-recyclable materials and energy/resources consumption.

### 3.6. Resin Properties and Performance

Characterisation across the reviewed literature predominantly relies on standardised protocols, although implementations vary. Tensile properties are most commonly evaluated using uniaxial tests in accordance with or ISO 527-2 [95]. Thermal stability is typically characterised via thermogravimetric analysis (TGA) following ISO 11358 [96], with T_5%_ values used as comparative metric. Glass transition temperature (T_g_) is most often determined through differential scanning calorimetry (DSC, ISO 11357) [97], though some studies employ dynamic mechanical analysis (DMA, ISO 6721) [98] for enhanced resolution. Chemical characterisation is mostly conducted through Fourier-transform infrared spectroscopy (FTIR), primarily to confirm epoxidation and monitor crosslinking reactions. Despite the widespread use of these methods, inconsistency in test conditions, curing parameters, and data reporting limit comparability.

#### 3.6.1. Physical Properties

**Figure 8:**
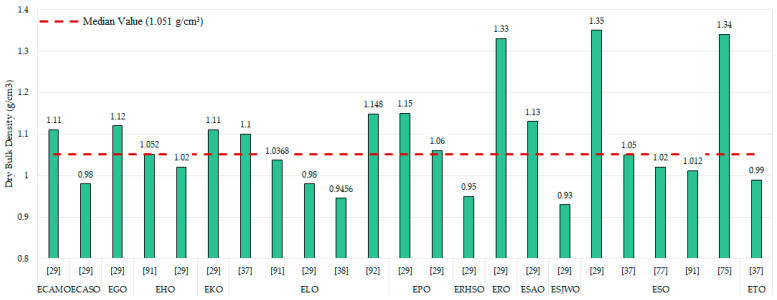
Bulk density data found in references [29,37,38,75,77,91,92].

Figure 8 presents dry bulk density values gathered from the studies analysed and grouped by type of oil used. The red dashed line marks the median value (1.05 g/cm^3^) of all samples. As can be seen, there is no direct correlation between type of oil and apparent density values. Lowest apparent density was achieved by St John’s Wort oil, (0.93 g/cm^3^) [29], whereas the highest value was obtained by Soybean oil 1.33 and 1.34 g/cm^3^ [29,75]. Typically, values range from 0.95 to 1.15 g/cm^3^, thus comprising 85% of the values. Values over this range could be due to the different resin formulations or chemical reagents used to develop the resin. Key parameters such as crosslinking density and curing time possibly affect resin properties, although there is not any mention nor reference across the reviewed literature. EEW data available for ESO in [77] (232 g eq^−1^) and [99] (242 g eq^−1^) are quite similar, as are the apparent density values obtained in both cases. A comparable trend was observed in studies involving ELO [29,99,100], where a OOC was also associated with increased apparent density. However, despite these parallels, the available data are still insufficient to establish a clear dependency between EEW/OOC and apparent density. Thus, it is not possible to establish a simple correlation between these values. Moreover, parameters such as chemicals used to elaborate the resin, curing degree, and crosslink density also have an enormous impact on these properties.

#### 3.6.2. Mechanical Properties

**Figure 9:**
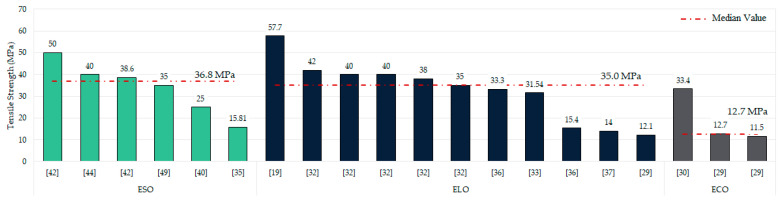
Tensile strength values according to type of oil used (MPa) [19,29,30,32,33,35,36,37,40,42,44,49].

A crucial characteristic of epoxidised oils-based resins is their mechanical properties. As previously noted, only 52.18% of the studies in this literature review conducted any form of mechanical characterisation. Figure 9 shows the tensile strength results, only including those studies whose values exceed 10 MPa to simplify the data presentation and to emphasise that values below this mark substantially limit the industrial or commercial viability of these resins.

As mentioned in the oil section, linseed and soybean are the most used oils. As can be seen, higher mechanical strengths are typically associated with already epoxidised linseed oil (ELO) and soybean oil (ESO) cured with various agents [32,42,44,45], then, soybean and linseed oils epoxidised in situ [19,29,36], and finally, the less common oils such as castor, corn, and camelina [29,30].

It is particularly noteworthy that the epoxidised soybean oil-based resin described by Sobhan et al. [40,46] stands out from the rest, achieving tensile strengths between 110 and 130 MPa using two different manufacturing methods. However, it should be mentioned that these resins were catalysed with acrylic acid and commercial curing agents. Overall, resins produced from ELO and ESO exhibit median tensile strengths of around 36.8 and 35 MPa, respectively, although some studies report higher values of up to 57.7 MPa (ELO) and 50 MPa (ESO) [19,42]. There are also cases where tensile strength ranges between 1 and 7.5 MPa for ESO-based samples [19,29,37,63,77,81,99,101,102] and ELO-based samples [82,89]. Other, less common oils—particularly recycled epoxidised soybean oil, pennycress, and mixed oils—have been reported to reach 4 MPa and 2.1 MPa tensile strengths, respectively, suggesting a promising and yet unexplored pathway for recycling edible oils [47,75,103].

**Figure 10:**
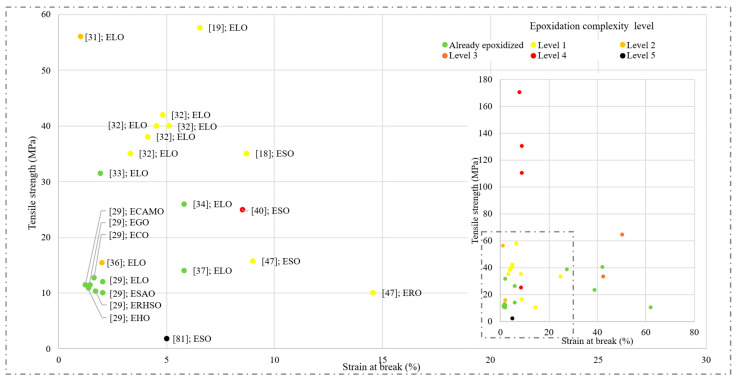
Tensile strength over strain at break results [18,19,29,31,32,33,34,36,37,40,47,81].

Subsequently, to contextualise resins’ performance, Figure 10 shows a dispersion chart on tensile strength (MPa) over strain at break (%), grouping results by an epoxidation complexity level set in the epoxidation processes section.

As in tensile strength bar chart, values exceeding 10 MPa are represented. Exceptionally, ESO-based resin developed by Saedi et al. is presented to show how complex epoxidation processes do not necessarily lead to a greater performance [81]. ELO and ESO-based resins are the most used oils across literature, although it must be noticed that these oils reported a wide range of both tensile and strain at break values. Already epoxidised oil-based resins from a study conducted by Di Mauro et al. were reported as stiff, moderate-strength samples, with 12 ±2 MPa tensile values using seven different oils. 2,2′-dithiodibenzoic acid (DTBA) was used as hardener, and three different commercial initiators—Imidazole (IM, 99%), 1-methylimidazole (1-MI, 99%), and N,N-dimethylbenzylamine (DMBA, 99%)—were tested, showing the impact of the hardener and catalysts in final performance [29]. Following the trend, a study conducted by Radojčić et al. using toluene and tris(pentafluorophenyl)borane (BCF) reported similar tensile values with a slightly higher strain at break (5.8%) [37]. These same reagent combinations were selected by Petrovic et al., increasing tensile strength up to 26 MPa and the same moderate stiffness (5.8%) [34].

Already epoxidised oil-based resins developed by Sangaletti et al. used synthesised diboronic ester dithiol (DBEDT), reporting tensile values around 23 MPa, although their strain at break increases proportionally up to 40%, making them very flexible [35]. Other already epoxidised oil-based resins increased this value, as per Grauzeliene et al., who through the UV curing method reported 31.54 MPa and 1.92% strain at break, thus proving the feasibility of this method on high-performance resins [33]. Similarly, ESO-based resins developed by Qian et al. [44] and Teijido et al. [45] used ethanol as a curing agent, reporting a higher strain at break (42% and 27.4%, respectively).

Mechanical strength increases as in situ epoxidation occurs, except for the sample developed by Tang et al., probably due to UV curing [47]. Samples belonging to level 1 of complexity in the epoxidation process are mostly based on linseed and soybean oils, due to their high stockage, together with the possibility of making comparisons with resins made from these already epoxidised oils. Thus, resin developed by Bach et al. using carboxyl-terminated poly(acrylonitrile-co-butadiene) (CTBN) as a hardener from 5 to 20 wt%. resulted in a maximum stress of 38–42 MPa, with moderate strain at break (4–5.1%) [32]. On the other hand, Lim et al. modified a commercial DEGB matrix by replacing 30 wt% with ESO, reporting 35 MPa. In this formulation, as ESO acts as a plasticiser, a higher strain at break (8.7%) was obtained, thus improving materials’ flexibility [18]. Within the level 1, resin elaborated by Liu et al. achieved 57.7 MPa tensile strength [19].

More complex levels of epoxidation, such as 2 and 3, reported similar values to those observed in level 1. ELO-based resin developed by Todorovic et al. reached 54 Mpa, although no values for strain at break were found [31]. Despite the good level 3 resins’ mechanical performance obtained by and Amrutha et al. (33.4 MPa, 64.3%) and Dominguez-Candela et al. (42.51 Mpa, 50.14%), epoxidised castor and safflower oil-based resins reported the greatest strain at break, thus reducing these resins’ potential applications [30,39]. Finally, the most complex epoxidation levels (4–5) reported distinct behaviour depending on the catalyst/hardener used. Whereas a study conducted by Sobhan et al. obtained values up to 170 MPa in certain formulations, resin developed by Saedi et al. required a more complex system, reporting low tensile strength values (1.8 MPa) and high strain at break [81].

**Figure 11:**
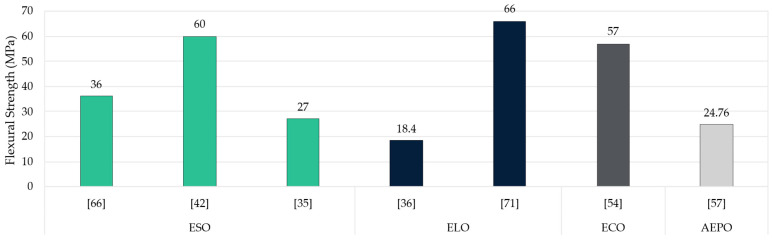
Flexural strength values grouped by type of oil used [35,36,42,54,57,66,71].

While the literature comprise sufficient tensile strength values, flexural strength is a less studied property, and its performance is surprisingly underexplored in bio-based resin studies as depicted in Figure 11. Epoxidised linseed (ELO), soybean (ESO), and castor (ECO) oils showed flexural strengths above 50 MPa, especially when already epoxidised oils and carefully optimised curing agents are used [42,54,71]. Still, around 30% of samples register values ranging from 2.76 to 7.1 MPa, which could limit their industrial utility [21,36,86,102]. Such discrepancies arise from differences in crosslink density, UV vs. thermal curing conditions, and the specific steps involved during in situ epoxidation. As a result, while the potential for strong bio-based materials is evident, the field lacks uniform testing protocols to compare outcomes consistently.

Current literature suggests that rather than the epoxidation process itself, the choice of curing agent, reaction conditions, and processing techniques have a greater impact on final strength. A study conducted by Bergoglio et al. used a UV-sensitive curing agent and photo initiators, thus highlighting how sample thickness and light penetration influence the crosslinking reaction [36]. On the other hand, in situ epoxidation often leads to greater variability than already epoxidised oils. This is because epoxy content and reaction can flow depending on reaction time and temperature [104]. As it happened with previous parameters analysed, the main constraint to further understand the behaviour of these materials is the lack of uniformity or standardisation in these resins’ studies.

**Figure 12:**
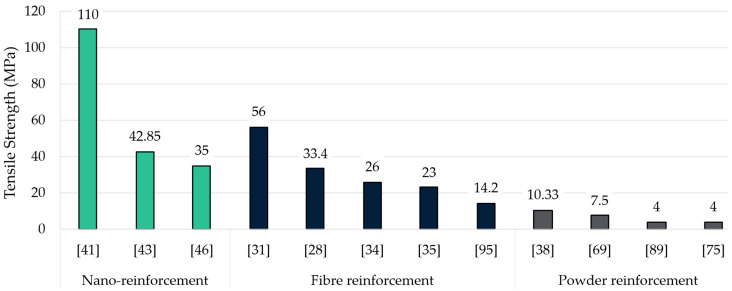
Reinforced bio-based resins tensile strength values grouped by type of reinforcement [28,31,34,35,38,41,43,46,69,75,89,95].

Although the literature reviewed was focused on epoxidation processes and resin characterisation, several studies considered reinforcing the resin, thus adding valuable extra data that have been also considered in this review. Figure 12 shows the tensile strength values collected. Reinforcements have been grouped according to the shapes they had, those being fibre, powder, and nano reinforcement.

As shown, reinforcing bio-based resins not always influence tensile strength, as values ranging from 4 to 10.33 MPa are found, especially in natural powder-shaped reinforced resins. To begin with, powder reinforcements had minimal impact on tensile strength. In fact, only one study obtained a value above 10 MPa, which used spruce bark (10.33 MPa) [38]. The addition of other wastes, such as sawmill wood chips [75], lignin powder [89], or newspaper waste [69], had less impact, and these values were even lower than other non-reinforced resins. Typically, powder-shaped additions have greater advantages in compressive strength rather than flexural strength.

Nevertheless, strength values significantly increase with fibre-shaped additions, especially when woven. Glass and carbon fibres are widely known and studied for their superior performance and versatility [105,106]. However, glass fibre-reinforced ELO-based samples reported low values (from 14.2 [95] to 26 MPa [34]) in this review, thus being far from the 70–100 MPa potentially achievable using commercial epoxy resin and this same fibre [107]. In between these two values is the ESO-based carbon fibre-reinforced sample reported by Sangaletti et al. (23 MPa [35]). Both oils are the most used in studies on epoxy resins based on epoxidised oil development. It is worth noting that, in this section, the highest tensile strength value was obtained using natural fibres such as hemp and epoxidised rice bran oil (33.4 MPa) [28]. This oil is a suitable alternative to conventional ELO and ESO. A potential reason for this oil to not be widely used is its low productivity (1 kg of rice bran per 10 kg of rice) [108]. Other fibre-shaped reinforced resins used shredded denims waste [109,110], glass fibre [70], flax fibre [111], and basalt fibre [85] although none of them performed mechanical testing [89].

Last, nano-reinforcement stands out for its potential to markedly improve mechanical properties without substantially increasing density in several fields. ESO resins modified with multi-walled carbon nanotubes and nanoclay [41] reach the highest tensile strength (110 MPa), thus highlighting the constructive interaction of nanoscale fillers and well-chosen epoxidised oils. Other nanoscale additives, such as CBNT and SiO_2_ (42.85 and 35 MPa, respectively) [43,46], also resulted in significant improvements. To achieve these results, a great and uniform dispersion is mandatory to successfully implement these reinforcements. As happened before, some studies using nano-reinforcement, such as graphene oxide [74], carbon nanotubes [112], and nanocellulose [113], did not perform any mechanical testing.

Future research should prioritise systematic mechanical characterisation including tensile strength, modulus, elongation at break, and flexural properties. To enable comparability, reporting parameters, such as epoxy equivalent weight (EEW), OOC, and crosslink density, is critical. The adoption of standardised mechanical testing protocols, such as ISO 527 [95] for tensile testing, is strongly recommended.

#### 3.6.3. Thermal Properties: Tg and Degradation Temperatures

**Figure 13:**
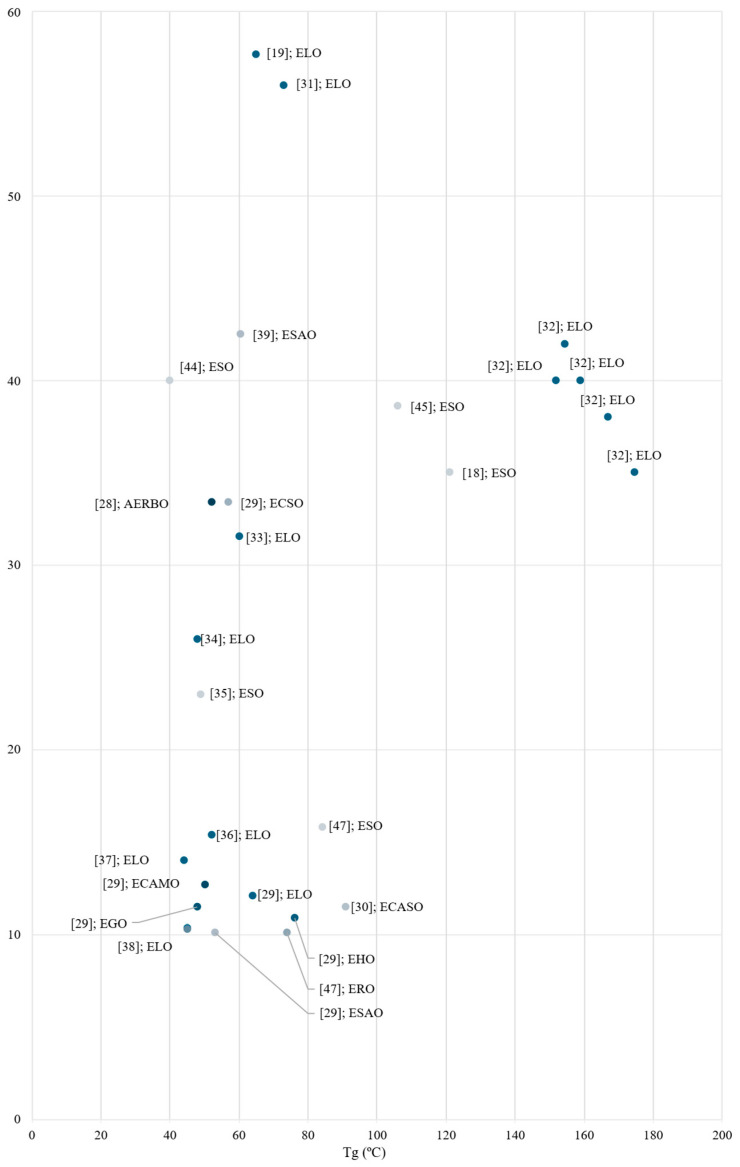
Tensile strength over glass transition temperature values [18,19,28,29,30,31,32,33,34,35,36,38,39,44,45,47].

Understanding the thermal stability of bio-based epoxy resins is crucial to set potential industrial applications and performance. In this review, key parameters such as glass transition temperature (T_g_) and thermal degradation temperature (T_5%_) have been gathered to determine the suitability of these resins in coatings, adhesives, and composites. Figure 13 and Table 4 show a T_g_ over the tensile strength dispersion chart, grouping samples by the type of oil used, to establish a correlation between values and performance.

As can be seen, there is not any direct linear correlation across the values found even when using the same oil. Typically, higher Tg values lead to a more rigid network, thus enhancing mechanical strength. However, values found in literature do not strictly support this fact. Samples with Tg values ranging 60 °C resulted in strength values over 100 MPa [41], whereas higher Tg values promoted strength values around 40–60 MPa [18]. Mechanical performance results found can be classified in three distinct groups as 0–20, 30–50, and >60 MPa. Then, Tg values are split through a 100 °C threshold.

Careful analysis revealed that ELO [29,36], ECAMO, ESAO, ECASO and EHO [29], and ESO and ERO [47]-based resins reported Tg values under 100 °C and relatively low tensile strength (<20 MPa). It is worth noting that most of the values within this range were obtained using the same epoxidation and curing process, which shows consistency and validates the procedure with several distinct oils. These values allow the materials to be used in low-mechanical requirement industrial applications, such as casings, buckets, and related products. Subsequently, another group of samples elaborated with diverse types of oil reported higher tensile strength (30–45 MPa) values with similar Tg. Less common oils, such as safflower (42.51 MPa) [39], rice bran [28], and chia seed [30] (33.4 MPa each) oils, are interesting alternatives to more common ELO [33] and ESO [44]-based resins, as they can perform similarly. Above these tensile strength values, two ELO-based resins stand out [19,31]. While a study conducted by Todorovic et al. epoxidised the oil in situ using a commercial Amberlite IR 120H to help the epoxidation process, a study conducted by Lyu et al., used already epoxidised oil. As expected, controlled elements such as already epoxidised oils and commercial products enhance mechanical performance.

Only three studies reported Tg values above 100 °C, though the mechanical performance is similar to those in the mid-range (20–50 MPa) [18,32,45]. These samples are ELO and ESO-based, thus highlighting the dispersion of Tg values and mechanical performance within samples using the same type of oil. Tensile strength ranged from 35 to 42 MPa in ELO-based resins [32], whereas ESO-based resins reported values closer to 35–38.3 MPa. As previously mentioned, Tg values can be quite different depending on factors such as peroxidation during epoxidation process and hardeners used to develop the resin. It is not possible to state any correlation between Tg values and mechanical performance, though most of the values found in this review reported values ranging from 40 to 100 °C.

**Figure 14:**
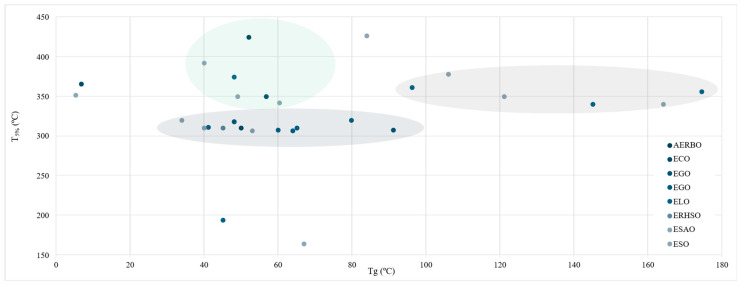
T_g_ values over T_5%_.

One of the most determinant thermal polymers’ parameters is thermal degradation. Figure 14 shows a dispersion chart on Tg over T_5%_ values. Compared to other materials, they have a relatively low thermal stability, thus limiting their range of applications [114]. Traditional organic polymers’ degradation temperature typically starts at temperatures ranging from 150 to 300 °C [115]. Within this review, all values but two presented a degradation temperature above 300 °C, with 426.3 and 425 °C being the highest temperature obtained [28,47], and 164 °C [41]. Both, the highest and the lowest temperature were obtained by ESO-based resins.

A mild upward trend can be observed where higher Tg often exhibit higher T_5%_. Typically, a denser crosslink—raising Tg values—also improves thermal stability. Three separate groups can be spotted according to their T_5%_. First, the biggest group of studies presented T_5%_ at 310 ± 10 °C, while Tg spanned from 34 to 91 °C. This group comprises samples elaborated with different oils where the epoxidation process was conducted in the study. Nevertheless, no oil stands out significantly as their properties were quite similar. Furthermore, a second group of studies, whose resins’ T_5%_ value was significantly higher than the first group (360 ± 20 °C), was conformed exclusively by ELO [32,68,71] and ESO [18,45,116]-based resin produced with already epoxidised oils. In this group, Tg values increased and spanned 96–174 °C.

Finally, a third disperse group of studies reported moderate Tg values and high thermal stability. Within this group, oils used are also diverse. AERBO [28] and ESO [44] samples reported the highest T_5%_, though their mechanical properties did not stand out (40 MPa) compared to other samples that reported lower Tg and higher tensile properties (65 °C-57.7 MPa) [19]. It is worth noting the singular combinations reported by Wu et al., and Gaina et al., whose ECO and ESO samples combined significantly low Tg (5.2–6.83 °C) with a high thermal stability, setting their T_5%_ at 352 and 366 °C, respectively [63,117]. On the other hand, a study conducted by Işık et al. and Dinu et al. reported moderate Tg with a low thermal stability [38,41].

To quantify these trends, Pearson correlation coefficients were calculated. A moderate positive correlation (r = 0.64) was found between OOC and tensile strength in the reviewed studies, suggesting that OOC is a relevant but not sole predictor of performance. Similarly, a mild positive correlation (r = 0.52) was observed between Tg and T_5%_, reflecting how increased crosslink density typically enhances both thermal stability and rigidity.

Of a particular importance is the fact that across the studies analysed, different epoxidised oil-based samples present quite different thermal properties, thus highlighting the absence of any correlation and an optimal procedure to ensure a proper thermal behaviour. Ultimately, no single approach ensures a reliable performance, thus emphasising the need for further, standardised, and systematic protocols to be applied.

### 3.7. Catalysts/Hardener’s Influence

As can be noticed, mechanical performance in epoxidised oil-based resins depends more on the choice of hardener and catalyst than on the type of oil feedstock itself. While base oils such as epoxidised linseed (ELO) or soybean oil (ESO) offer the starting network, it is the curing system—the combination of crosslinkers and accelerators—that truly dictates how strong, stiff, or flexible the final resin becomes. To better understand this influence, we must explore not just their mechanical outcomes, but also the sustainability, thermal behaviour (T_g_), and material density of these chemical components. These hidden factors explain why two resins with similar oils perform so differently in real-world conditions.

To begin, a comparison between aromatic and aliphatic crosslinkers is a distinction that offers a clear divide in mechanical performance. Aromatic systems, such as 2,2′-dithiodibenzoic acid (DTBA) or Aradur 956, both rich in rigid benzene rings, consistently produce high T_g_, high stiffness, and moderate to high tensile strength. In the study by Di Mauro et al. [29], ESO cured with DTBA and catalysed by imidazole derivatives achieved tensile strengths of up to 12.7 MPa, paired with glass transition temperatures between 17 and 91 °C. These stiff networks owe their strength to the rigidity of the aromatic backbone and high functionality of the hardener, which reduces the flexibility of polymer chains and reinforces the three-dimensional structure. However, these same structures are synthesised through complex petroleum-based processes, thus carrying moderate toxicity and poor biodegradability, which compromises their environmental credentials.

In contrast, aliphatic polycarboxylic acids, such as citric acid, succinic acid, or adipic acid, offer greater sustainability, as they can be derived from biomass fermentation or plant oils with low toxicity and energy input. These systems often produce more flexible networks, with lower T_g_ values typically in the 50–100 °C range. For example, citric acid-cured resins reported by Quispe et al. [82] achieve tensile strengths around 8.8 MPa, but strain at break values of up to 50%, indicating a highly ductile material. These softer backbones are excellent for applications requiring elasticity or impact resistance, but their lower stiffness and reduced thermal stability may limit their use in structural components or high-temperature environments.

Subsequently, there are compelling middle-ground systems that balance strength, flexibility, and sustainability. The UV-cured acrylate formulations described by Vita et al. [36] stand out for their clever use of iso-bornyl methacrylate (IBOMA) and THFA, two monomers with moderate rigidity and bio-based potential. When paired with a high efficiency photoinitiator, these resins achieved tensile strengths up to 33.3 MPa and glass transition temperatures near 96 °C. While slightly less sustainable than the polyacid-based systems due to the photoinitiators’ fossil-derived origin, these networks demonstrate how tuning both the chemical structure and curing mechanism can yield resins with industrial-grade performance while maintaining moderate environmental impact.

The role of catalysts is just as critical. Systems that incorporate imidazole derivatives—such as 1-methylimidazole or 1-imidazole—often show superior network homogeneity and conversion efficiency. These nucleophilic catalysts activate the epoxy-anhydride reaction more efficiently than traditional amines or acids, resulting in higher tensile strength and higher T_g_. This catalytic system stands in contrast to more traditional amine curing systems (e.g., DETA), which, while still effective, often result in lower T_g_, less densely crosslinked networks, and greater variation in mechanical properties.

The synergistic action of acrylic acid (a multi-functional monomer introducing additional crosslink points) and optimised commercial hardeners dramatically boosts the strength of the network. However, this formulation also comes with trade-offs: acrylic monomers such as HEMA and MAA are fossil-derived and energy intensive, and their use may involve moderate toxicity and end-of-life disposal concerns. These examples illustrate that mechanical excellence often comes at a cost to sustainability, unless future work develops bio-based analogues or improves LCA data to justify their use.

On the other hand, peroxide-cured ESO resin exhibits infinite strain at break but low tensile strength (~4 MPa) [75]. The likely cause is poor crosslink density due to incomplete peroxide initiation or low reactivity of the resin, leading to a polymer network that can stretch endlessly but lacks cohesive force. Conversely, the thiol-ene network based on DBEDT from Sangaletti et al. [35] reaches 23 MPa tensile strength and 40% strain at break, aided by its sulphur-rich structure and well-distributed crosslinking. This system combines a relatively high density (~1.4 g/cm^3^), moderate T_g_, and excellent elasticity, illustrating how thiol-based systems can offer both toughness and flexibility, although with less ideal environmental profiles due to sulphur and aromatic content.

Aromatic hardeners give excellent strength and thermal stability but are less sustainable. Aliphatic and bio-derived acids improve flexibility and biodegradability but may compromise strength. UV curing delivers high performance with low energy input, yet may require photoinitiators that are hard to recycle or replace. Imidazole catalysts enhance crosslinking efficiency but stem from oil-based sources.

From an environmental standpoint, EVO-based resins offer significant advantages, including the use of renewable resources, the potential for biodegradability (especially with polyacid hardeners), and lower carbon footprints. Resins cured with bio-derived agents such as citric or tartaric acid avoid the hazardous by-products associated with fossil-derived crosslinkers, such as bisphenol A. Furthermore, simpler epoxidation processes, such as Prileschajew reactions with green acids, minimise both energy input and chemical waste, enhancing overall sustainability.

Therefore, future formulation strategies must aim to combine the best of these systems: selecting bio-based monomers with functional groups that enable high crosslink density, using efficient, low-toxicity catalysts, and optimising cure conditions to achieve desired mechanical and thermal properties. Only by integrating green chemistry with performance metrics can we push forward the development of bio-based resins that are not only effective, but also sustainable, scalable, and responsible.

### 3.8. Complementary Tests and Applications

Fire resistance is one of the polymeric products’ weak points and one of the most decisive when thinking about these resins’ potential applications. However, only four studies addressed this issue. In two of their studies, Cabo et al. worked on the modification of a commercial resin matrix with epoxidised corn oil to evaluate the cone calorimeter and the decomposition time of this material, reporting an improvement in the heat release ratio (HRR) of 17.07% with respect to traditional resins [24,118]. Li et al. and Wang et al. performed these tests on resins made purely from epoxidised honokiol and soybean oils, in which additions of wood residues and a synthesis of furan-2-ylmethyl diphenylphosphinate reduced the HRR of the samples and slowed down the combustion process [59,102]. The characterisation and improvement of the fire resistance of these resins is positioned as a great opportunity to be explored in future studies, as the existing information is scarce.

One of the most relevant aspects found in this review is the lack of studies on these materials’ life cycle assessment (LCA) and recyclability. Only one study conducted a full cradle-to-gate life cycle analysis, comparing sucrose soyate (ESS)-based resins with commercial systems [111]. Other studies worked on the recyclability of resins by grinding and adding old resins to new matrices, thus reducing raw material consumption [119]. Surprisingly, thermal stability remains within the same range of values, and their mechanical performance showed good compatibility, thus showing a pathway still to be explored [35,90,120].

Among the studies reviewed, most did not specify any application for the resin or epoxidised oil developed. However, those that did highlighted its potential use in various fields. One notable application is in 3D printing, where the resin can be cured using different methods, such as UV light or high-temperature curing, offering versatility in fabrication processes [30,42,93,121,122]. Additionally, one of the most common uses of these resins is in the production of packaging films for medical or commercial purposes, where their mechanical properties proved them valid and sustainable in packaging applications [39,40,55,65,74,123].

## 4. Conclusions and Future Perspectives

Epoxidised oils, through their epoxidation processes and resin synthesis, represent a promising alternative to conventional epoxy systems. Their abundant availability, broad range of applications, and the significant potential for further research in material characterisation and development provide compelling reasons to pursue deeper investigation into these materials. Accordingly, this literature review led to the following conclusions:

ELO and ESO are the most used oils in either epoxidation processes or resin elaboration. The reason to be widely used is because only these two oils can be found already epoxidised, thus saving both cost and time while developing the resins. Expectedly, already epoxidised oils and in situ epoxidised oils performed differently, although there is no correlation between the final properties due to the wide range of reagents used in resins elaboration. Analysing the in situ epoxidised oils, a clear correlation was observed between OOC, and resin performance can be spotted. OOC resulted in a normal distribution, where 5–7% showed the best balance, as values behind and above this range showed an excessive crosslink density that affected their microstructure, thus increasing brittleness. As happened with OOC content, high Tg values did not lead to superior tensile strength. Rather than a high value, a moderate Tg enhanced mechanical performance. This pattern is also found in thermal degradation temperature. The data show a better performance in balanced T_g_-T_5%_ values (T_g_ 50–70; T_5%_ 300–350).

After analysing the epoxidation processes, complex multi-step methods do not necessarily lead to a better performance compared to easier processes. Moreover, the use of hazardous and polluting chemical reagents would not help moving on to sustainable and greener resins. Most of the studies proved that the basic Prileschajew reaction epoxidised oils to report similar mechanical properties while reducing the need for hazardous reagents, thus making these resins easier to elaborate and work with. Numerous chemical reagents used to develop the resin reported dispersed results. It was not possible to determine a good formulation to ensure a reliable performance as the different variables (oil used, composition, reagents, and curing/post curing time) had different impacts in the final properties.

Only 38% of the studies analysed in this review performed at least one mechanical characterisation test. With tensile and flexural strength being the most common techniques needed to understand material’s behaviour and consider potential applications, a greater effort should be put forth to include this property in the studies. The highest tensile strength obtained (ELO-based resin, 170 MPa) demonstrates the suitability of these materials to be applied in high-requirement applications. A slight link between OOC and tensile strength was observed, thus highlighting the importance of a balanced epoxidation approach.

The mechanical performance of epoxidised oil-based resins is fundamentally determined by the selection of hardeners and catalysts, far more than the type of vegetable oil used. Aromatic anhydrides such as 2,2′-dithiodibenzoic acid (DTBA) and tetrahydromethylphthalic anhydride (Aradur 956), when combined with nucleophilic accelerators such as 1-methylimidazole (1-MI) or N,N-dimethylbenzylamine (BDMA), yield high-performance resins with tensile strengths exceeding 50 MPa and glass transition temperatures well above 300 °C. These formulations should be prioritised for structural or high-temperature applications. On the other hand, UV-curable acrylate systems using methallyl alcohol (MAA) and HEMA crosslinked with TMPTA and initiated by Darocur 1173, also demonstrated excellent strength (up to 33 MPa) and fast curing under ambient conditions, making them highly suitable for coatings and lightweight composites.

Moving toward sustainable high-performance resin systems requires a careful compromise between green chemistry and functional output. While fully bio-based acids, such as citric acid, tartaric acid, and itaconic acid, offer low toxicity, biodegradability, and reduced environmental impact, they tend to produce more flexible and less stiff networks, with tensile strengths typically below 10 MPa. However, combining these with multifunctional amines, such as diethylenetriamine (DETA), or using dual-curing strategies with UV activation and thermal post-curing could significantly improve performance. Future research should focus on hybrid formulations that exploit the tunability of bio-derived acids while leveraging the high reactivity of imidazole-based catalysts or acrylate crosslinkers to enhance both mechanical strength and environmental profile.

From the reinforced resins across the literature, nano-reinforcement showed the best performance in almost all key parameters studied, such as tensile strength and thermal stability. This aligns with the current trends in other materials. Nevertheless, it is still to be explored, as only few studies used these reinforcing elements. Fibre-reinforced composites are commonly studied with conventional systems. In this review, natural fibres such as hemp outperformed synthetic fibres in tensile strength, suggesting a better interaction when combining two natural elements.

There are several inconsistencies in reporting fundamental chemical properties across the studies analysed. This fact makes direct comparison challenging. Although standardised studies would not fix the problem, reporting certain basic data would help authors compare and improve the quality of the knowledge.

Additionally, while many studies focus on optimising epoxidation and resin formulation, comprehensive life cycle assessments (LCAs) and environmental impact studies are largely absent. Integrating these analyses into future research would provide a clearer understanding of the true sustainability potential of these materials. Future work should address the following:

- Developing standardised testing protocols to enable better comparisons between studies.
- Investigating alternative bio-based epoxidation agents with lower toxicity and environmental impact.
- Enhancing nano-reinforcement strategies to improve mechanical performance while maintaining biodegradability.
- Conducting detailed life cycle assessments to evaluate the long-term feasibility of bio-based epoxy resins in industrial applications.

By addressing this challenges, future research will work towards high-performance, sustainable epoxy resins able to replace conventional fuel-based systems. Table 5 pictures the main problems, impact and proposed solutions found in this review. Future work should consider the items stated and add that valuable information to standardise the results.

## Figures and Tables

**Table 1:** Epoxidised oil’s main properties found in litetature.

Epoxidised Oil Used	Ref	Iodine Value (IV)	Theoretical Oxirane Content (%)	Flexural Strength (MPa)	Flexural Modulus (GPa)	Tensile Strength (MPa)	Tensile Modulus (GPa)	Strain at Break (%)	T_onset_ (°C)	T_g_ (°C)
**AERBO**	[28]	6.3	5.30	26.6	-	33.4	-	25	425	52
**ECAMO**	[29]	-	5.29	-	-	12.7	0.825	1.62	310	50
**ECASO**	[29]	-	2.85	-	-	11.5	0.815	1.23	308	91
**ECSO**	[30]	197	6.70	-	-	33.4	2.893	64.3	350	56.8
**EGO**	[29]	-	4.94	-	-	11.5	0.804	1.42	318	48
**EHO**	[29]	-	6.09	-	-	10.9	0.811	1.36	310	76
**ELO**	[19]	-	6.23	-	-	57.7	1.632	6.52	310	65
	[31]	-	-	-	-	56	5.2	-	-	73
	[32]	-	-	-	-	42	-	4.8	-	154.5
	[32]	-	-	-	-	40	-	4.5	-	158.9
	[32]	-	-	-	-	40	-	5.1	-	151.9
	[32]	-	-	-	-	38	-	4.1	-	166.8
	[32]	-	-	-	-	35	-	3.3	356.56	174.5
	[33]	-	-	-	-	31.54	2.45	1.92	308	60
	[34]	-	9.50	-	-	26	0.791	5.8	375	48
	[35]	-	5.60	27	0.85	23	0.43	39	350	49
	[36]	-	-	7.1	0.523	15.4	0.505	4.7	-	52
	[37]	-	9.40	-	-	14	0.35	5.8	-	44
	[29]	-	5.61	-	-	12.1	0.83	2.04	307	64
	[38]	-	-	-	-	10.33	0.178	61.87	194	45
**ESAO**	[39]	-	5.70	-	-	42.51	1.931	50.14	341.91	60.3
	[29]	-	4.93	-	-	10.1	0.771	2.04	307	53
**ESO**	[40]	-		-	-	170	-	8	-	-
	[40]	-		-	-	130	-	9	-	-
	[40]	-		-	-	110	-	9	-	-
	[41]	-	-	-	-	110	5.6	0.9	164	66.94
	[42]	-	-	60	1.6	50	-	13	-	-
	[43]	54.78	-	-	-	42.85	-	4.84	218.62	-
	[44]	-	-	-	-	40	-	42	392.72	40
	[45]	-	6.40	-	-	38.6	0.232	27.4	378	106
	[18]	-	6.90	-	-	35	1	8.7	350	121
	[46]	54.78	-	-	-	35	-	4	-	-
	[40]	-	5.5	-	-	25	-	8.5	360.3	-
	[47]	-	7.34	-	-	15.81	0.259	8.99	426.3	84

**Table 2:** Chemical compounds used.

Toxicity Level	Epoxidation Agent	Times Used	Formula	Role	Reactivity
1	Acetylcholine	1	C_7_H_16_NO_2_	Reagent/additive	Reactive
1	Sodium bicarbonate	4	NaHCO_3_	Base/neutralising agent	Reactive
2	Amberlite IR 120H	28	-	Acid catalyst	Stable
2	Choline chloride	2	C_5_H_14_ClNO	Component for DES	Stable
2	Citric acid	1	C_6_H_8_O_7_	Acid catalyst	Stable
2	Magnesium sulphate	3	MgSO_4_	Drying agent	Stable
2	Sodium acetate	1	C_2_H_3_NaO_2_	Buffer/reagent	Stable
3	Benzyl triethylammonium	5	C_13_H_22_N	Phase transfer catalyst	Stable
3	Fumaric acid	1	C_4_H_4_O_4_	Acid catalyst/reagent	Stable
3	Oxalic acid	3	C_2_H_2_O_4_	Acid catalyst	Reactive
4	Formic acid	13	CH_2_O_2_	Oxidant	Reactive
4	Glacial acetic acid	20	C_2_H_4_O_2_	Acid catalyst and solvent	Reactive
4	Hydrogen peroxide	34	H_2_O_2_	Oxidant	Reactive
4	Phosphoric acid	1	H_3_PO_4_	Acid catalyst	Reactive
4	Sodium hydroxide	4	NaOH	Base	Reactive
4	Triethylenetetramine	1	C_6_H_18_N_4_	Curing agent/catalyst	Reactive
5	Epichlorohydrin	4	C_3_H_5_ClO	Primary epoxidising agent	Reactive
5	Hydrogen bromide	1	HBr	Acid catalyst	Reactive
5	Phenol formalin solution	1	C_7_H_8_O	Crosslinker/reactant	Reactive
5	Sulfuric acid	13	H_2_SO_4_	Acid catalyst	Very reactive
5	Toluene	5	C_7_H_8_	Solvent	Stable

**Table 3:** Top 10 tests performed and topic distribution.

1	FT-IR—74.42%	Chemical properties	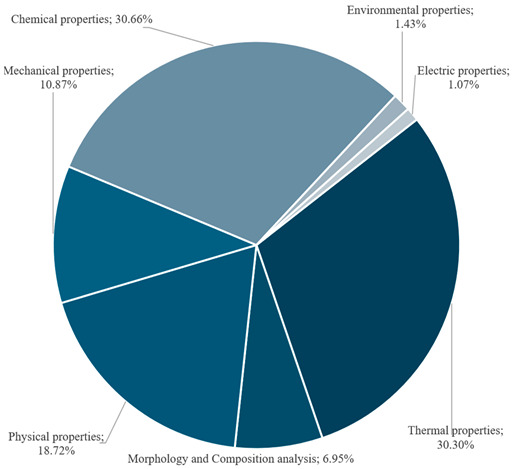
2	TGA—68.60%	Thermal properties	
3	DSC—62.79%	Thermal properties	
4	DMTA—50.0%	Thermal properties	
5	NMR—40.70%	Chemical properties.	
6	Tensile—38.37%	Mechanical properties	
7	SEM—32.56%	Morphology and comp.	
8	Flexural—19.77%	Mechanical properties	
9	Hardness—16.28%	Physical properties	
10	Gel content—15.12%	Chemical properties	

**Table 4:** Tg and tensile strength values.

Epoxidised Oil Used	Ref	T_g_ (°C)	Tensile Strength (MPa)
ELO	[19]	65	57.7
ELO	[31]	73	56
ESAO	[39]	60.3	42.51
ELO	[32]	154.5	42
ELO	[32]	158.9	40
ELO	[32]	151.9	40
ESO	[44]	40	40
ESO	[45]	106	38.6
ELO	[32]	166.8	38
ELO	[32]	174.5	35
ESO	[18]	121	35
AERBO	[28]	52	33.4
ECSO	[30]	56.8	33.4
ELO	[33]	60	31.54
ELO	[34]	48	26
ESO	[35]	49	23
ESO	[47]	84	15.81
ELO	[36]	52	15.4
ELO	[37]	44	14
ECAMO	[29]	50	12.7
ELO	[29]	64	12.1
ECASO	[29]	91	11.5
EGO	[29]	48	11.5
EHO	[29]	76	10.9
ELO	[38]	45	10.33
ERHSO	[29]	45	10.3
ERO	[47]	73.9	10.12
ESAO	[29]	53	10.1

**Table 5:** Main problems identified and proposed solutions.

Problem	Impact	Proposed Solution
Lack of standardised mechanical testing	Inconsistent comparisons	Use of, ISO 527 [95] for tensile/flexural testing
Variable epoxy content reporting	Inaccurate correlation with performance	Require OOC and EEW reporting
Toxic/hazardous hardeners	Limits eco-certification and safety	Favor bio-based acids (e.g., citric, tartaric)
Limited reinforcement dispersion	Reduced mechanical gains	Improve mixing (ultrasound, shear) and surface treatments

## Abbreviations

FT-IR	Fourier Transform Infrared Spectroscopy
NMR	Nuclear Magnetic Resonance
TGA	Thermogravimetric Analysis
DSC	Differential Scanning Calorimetry
DMTA	Dynamic Mechanical Thermal Analysis
SEM	Scanning Electron Microscopy
LCA	Life Cycle Assessment
OOC	Oxirane Oxygen Content
EEW	Epoxy Equivalent Weight
IV	Iodine Value
Tg	Glass Transition Temperature
T5%	Temperature at 5% Weight Loss
CBNT	Carbon-Based Nanotubes
Tonset	Onset Temperature
DES	Deep Eutectic Solvent
